# Appetite Regulation of TLR4-Induced Inflammatory Signaling

**DOI:** 10.3389/fendo.2021.777997

**Published:** 2021-11-24

**Authors:** Yongxiang Li, Qingyan Jiang, Lina Wang

**Affiliations:** ^1^ Guangdong Provincial Key Laboratory of Animal Nutrition Control, South China Agricultural University, Guangzhou, China; ^2^ National Engineering Research Center for Breeding Swine Industry, College of Animal Science, South China Agricultural University, Guangzhou, China; ^3^ Children’s Nutrition Research Center, Department of Pediatrics, Baylor College of Medicine, Houston, TX, United States

**Keywords:** TLR4, inflammation, hypothalamus, dopamine system, SFAs

## Abstract

Appetite is the basis for obtaining food and maintaining normal metabolism. Toll-like receptor 4 (TLR4) is an important receptor expressed in the brain that induces inflammatory signaling after activation. Inflammation is considered to affect the homeostatic and non-homeostatic systems of appetite, which are dominated by hypothalamic and mesolimbic dopamine signaling. Although the pathological features of many types of inflammation are known, their physiological functions in appetite are largely unknown. This review mainly addresses several key issues, including the structures of the homeostatic and non-homeostatic systems. In addition, the mechanism by which TLR4-induced inflammatory signaling contributes to these two systems to regulate appetite is also discussed. This review will provide potential opportunities to develop new therapeutic interventions that control appetite under inflammatory conditions.

**Graphical Abstract:**
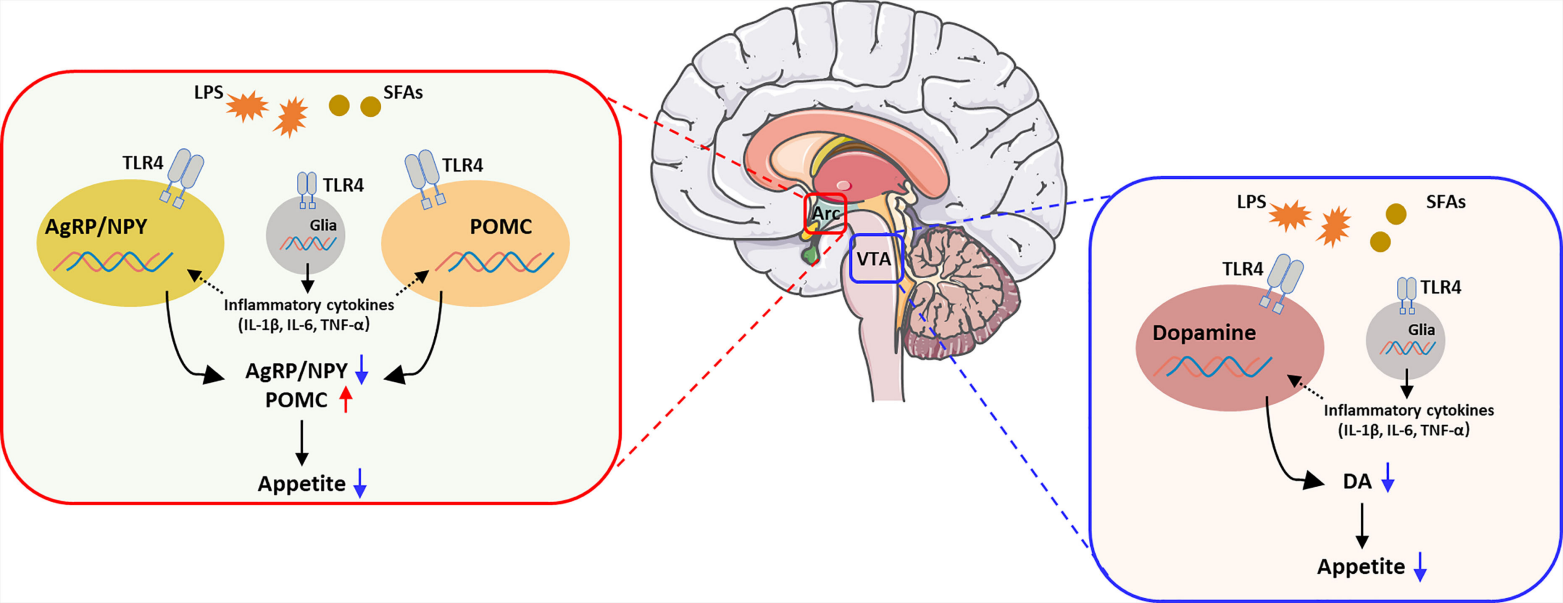
The activation of TLR4 in the regulation of appetite. Brain image was provided by smart.servier.com.

## Introduction

Appetite plays an essential role in the regulation of energy balance. Metabolic requirements must be met by obtaining and consuming food. In this process, the brain plays a vital role in regulating energy metabolism. At the most basic level, the regulation of appetite by the central nervous system (CNS) is divided into two types: homeostasis and non-homeostasis. The homeostatic system controls feeding to resolve general energy deficiencies or meet other types of metabolic needs. However, the non-homeostatic system, also called the reward system, is typically driven by palatable foods with high fat or sugar contents. Food intake results from various physiological and behavioral processes that control hunger, satiety, and reward systems. Recently, the regulation of feeding by inflammatory signals such as TLR4 has been reported frequently. TLR4 is a transmembrane pattern recognition receptor that recognizes molecular patterns related to injury and pathogens (1, 2). TLR4 is expressed on cells of the innate immune system in the CNS, such as endothelial cells, microglia, and some astrocytes, as well as adult neurons (3, 4). Pattern recognition receptors have been extensively developed to recognize a wide variety of pathogen-related molecular patterns (PAMPs) associated with microbial pathogens or cell signals of danger or stress. Ligands bind to TLR4 and its accessory molecules, such as myeloid differentiation protein 2 (MD2) and cluster of differentiation 14 (CD14), to activate downstream intracellular signaling pathways, thus producing and releasing proinflammatory and neuroexcitatory mediators *via* MyD88-dependent or MyD88-independent intracellular pathways (5). Therefore, TLR4-induced inflammatory signaling provides a mechanistic link between the hypothalamus, mesolimbic dopamine (DA) system and appetite (6).

Here, we will focus on current insights into the regulation of appetite by TLR4-induced inflammatory signaling in the hypothalamus and mesolimbic DA system. In addition, we also explored recent publications to investigate the activation of TLR4-induced inflammatory signaling and its effect on appetite.

## TLR4-Induced Inflammatory Signaling in the Hypothalamus Affects the Appetite Process

### Energy Homeostasis and Inflammatory Signaling

**Figure 1:**
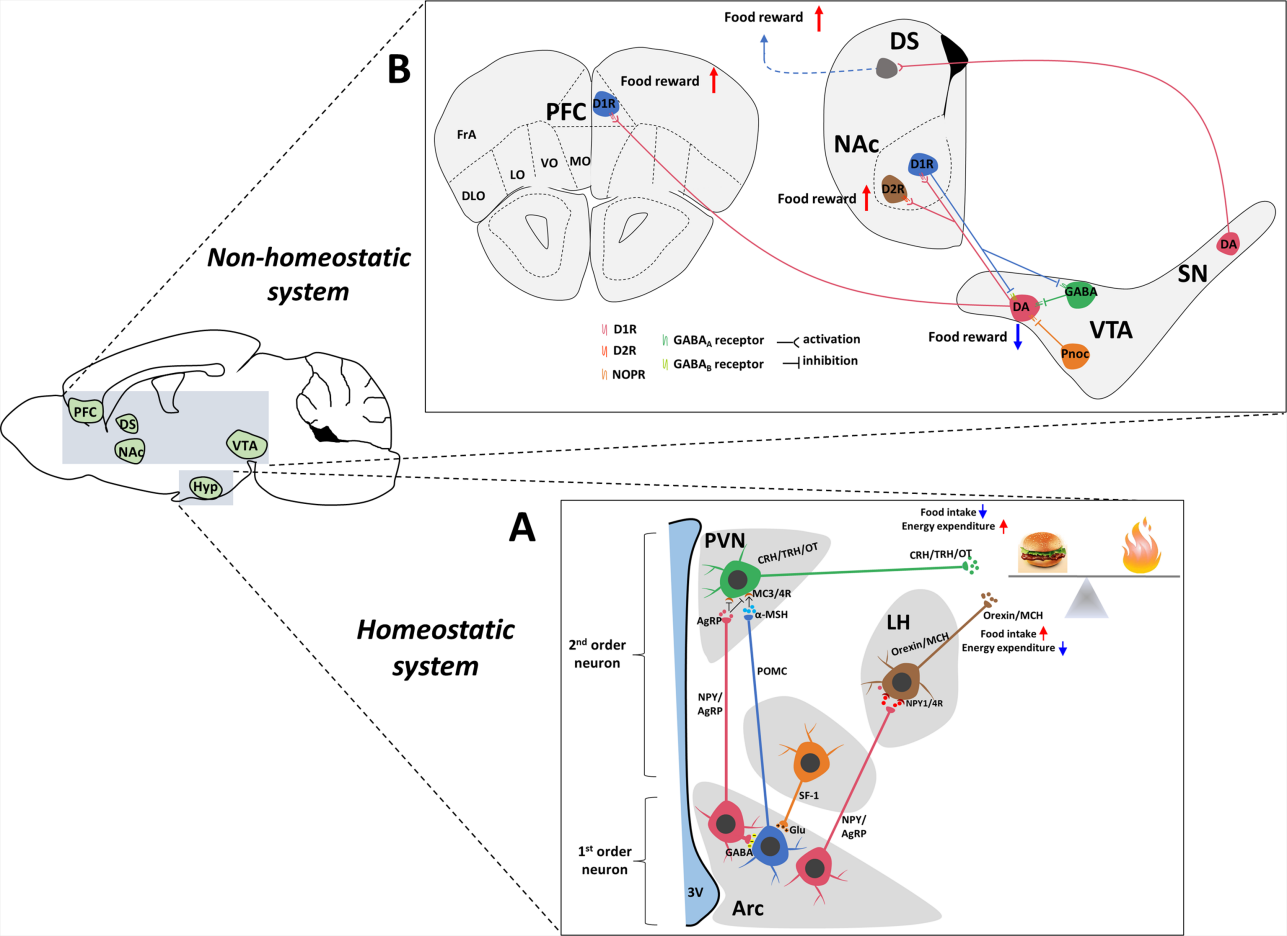
Central neuronal circuits involved in homeostatic and non-homeostatic systems. **(A)** Hypothalamic regulation of energy homeostasis. The Arc of the hypothalamus contains two groups of neurons: NPY/AgRP and POMC neurons. These neurons secrete orexigenic AgRP/NPY or anorexigenic POMC to the second-order neurons in the PVH and LH. In the PVN, neurons produce OT, TRH and CRH to decrease food intake. Neurons in the LH produce ORX and MCH to increase food intake. AgRP is an antagonist of anorectic MC4R expressed on neurons within the PVN. In the Arc, a local circuit exists in which POMC neurons receive GABAergic (inhibitory) input from NPY/AgRP neurons. The SF-1 nerve located in the VMH innervates POMC/CART neurons to enhance the anorexia function of POMC/CART neurons and is critically involved in the regulation of energy homeostasis. **(B)** The dopamine system, which contains connections between the midbrain and forebrain. The reward pathway includes dopaminergic neurons in the ventral tegmental area that project to the NAc and PFC. Dopamine, which is released from dopaminergic neurons in the VTA, binds to D1R or D2R in the NAc and D1R in the PFC, which contribute to food reward. In turn, D1R also inhibits DA and GABAergic neurons in the VTA through different GABA receptors to form negative feedback regulation. The VTA also contains GABAergic cells and Pnoc neurons that project to DA neurons to inhibit DA activity. In addition, DA neurons located in the SN also project to the DS, which potentially increases food reward behavior. Hyp, hypothalamus; Arc, arcuate nucleus; LHA, lateral hypothalamus area; PVN, paraventricular nucleus of the hypothalamus; VMH, ventromedial hypothalamus; 3V, 3rd ventricle; VTA, ventral tegmental area; NAc, nucleus accumbens; PFC, prefrontal cortex; DS, dorsal striatum; SN, substantia nigra; AgRP, agouti-related peptide; NPY, neuropeptide Y; POMC, proopiomelanocortin; Glu, glutamate; GABA, gamma-aminobutyric acid; MCH, melanin-concentrating hormone; α-MSH, α-melanocyte-stimulating hormone; CRH, corticotropin-releasing hormone; TRH, thyrotropin-releasing hormone; OT, oxytocin; DA, dopamine; D1R, dopamine 1 receptor; NOPR, nociceptin opioid peptide receptor.

Appropriate energy homeostasis results from a delicate balance between energy intake and expenditure. Substantial evidence indicates that arcuate nucleus (Arc) has emerged as a key regulator of energy homeostasis (7). The key to this process is two sets of interconnected Arc neurons (**Figure 1**). As described below, orexigenic neuropeptide Y (NPY)/agouti gene-related protein (AgRP) neurons and anorexigenic proopiomelanocortin (POMC) neurons play opposite but coordinated roles in controlling food intake. In addition, neurons that are distributed in the paraventricular hypothalamic nucleus (PVH), lateral hypothalamus (LH) and ventromedial hypothalamus (VMH) are also involved in regulating energy homeostasis (8–10). In most cases, our bodies are constantly adapting to changes in the external environment that are essential for survival. Normally, the existence of the immune system always maintains the normal function of organs. However, when the immune system is activated by pathogens or other stimuli, typical symptoms such as fever, anorexia and pain occur. Two types of proinflammatory responses to immune system activation have been identified: (1) acute inflammation, such as a bacterial infection that lasts for a few minutes to several hours, and (2) chronic inflammation, such as obesity induced by a high-fat diet (HFD), cancer, and chronic obstructive pulmonary disease (COPD), which ranges from days to years. Although peripheral inflammation plays a key role in regulating energy metabolism and appetite (11), central production of inflammatory mediators is more important than peripheral production in regulating appetite and body weight. Recent discoveries have indicated that the brain contains lymphatic vessels and thus forms a lymphatic drainage system (12). The discovery that lymphocytes are useful to examine diseases related to the CNS and the immune response in the break further overcomes the misconception of the brain as an immune-isolated organ (13).

Although we understand how to achieve the balance between pathology and physiology, the effect of inflammatory processes on homeostasis remains inadequate. Energy homeostasis and the immune system have many coordinated responses. Disease syndrome is a coordinated response by the brain to help the immune system combat infection, which is accompanied by typical symptoms such as fever, anorexia and pain. For example, fever is an increase in the core body temperature that enhances immune cell function to eliminate pathogens. In addition, sleepiness and achiness decrease energy expenditure by decreasing locomotor activity. Last, the reduction in food intake decreases glucose supply available to the infectious agent (14). Over the past few decades, research has highlighted the key role of proinflammatory cytokines such as interleukin-1β (IL-1β), interleukin-6 (IL-6), and tumor necrosis factor-α (TNF-α) in controlling energy homeostasis in anorexic-cachexia syndrome. Most studies support the hypothesis that the hypothalamus plays a key role in reducing food intake and the development of illness with inflammation.

### How Does the Inflammatory Signaling Decrease Appetite to Maintain the Energy Balance?

Chronic diseases, such as cancer, disrupt these very primitive and coordinated responses. In cancer-induced anorexia, central sensors of energy homeostasis are rapidly associated with increased energy expenditure and decreased food intake. The aim of this process is to reduce the glucose supply to cancer cells. However, rapidly proliferating cancer cells have lost their oxidative glycolysis capabilities, and thus they can develop a chronic malnutrition state (15). Notably, most of these causes are usually associated with “high-grade” inflammation. Even if the pathological states are benign and short term, they are also highly inflammatory and associated with decreased appetite or “anorexia”. Anorexia is a result of the classic defense of an organism against infections, also known as “sickness behavior”. (16, 17). The production of proinflammatory cytokines is induced by infectious factors, and higher TNF-α, IL-1β and IL-6 levels affect the surrounding organs to induce disease behavior and inflammation-related anorexia, which participate in the induction of disease behavior (18). Hypothalamus cannot regulate energy balance under increasing energy expenditure and chronic inflammation (19). Neuroinflammation and hypothalamic signal transduction were affected by chronic inflammation induced by cancer (19), HIV (20), COPD (21), and heart failure (22). This inflammatory response in the hypothalamus is elicited by elevated plasma cytokine levels entering the brain, as several cytokines are able to cross the blood–brain barrier (BBB), including TNF-α (23), IL-6 (24), IL-1α (25) and IL-1β (26). In addition, many receptors for these proinflammatory cytokines are expressed in the hypothalamus (27). Hypothalamic neurons in the Arc sense peripheral circulating factors, including cytokines, from the adjacent median eminence (ME), without protection of the blood–brain barrier.

Indeed, a common phenomenon appears to be that hypothalamic microglia, astrocytes and macrophage-like cells are activated in the hypothalamus in response to chronic inflammatory diseases (28, 29). Chronic administration of cytokines reproduces the characteristics of anorexia syndrome (30–32), while its development is inhibited by blocking the signaling of one of these cytokines, such as TNF-α, using neutralizing antibodies (33, 34). In animal models of inflammatory anorexia, interfering with inflammatory mediators reduces hypothalamic inflammation and prevents weight loss. For example, inhibiting the action of adenosine monophosphate protein kinase (AMPK) in the hypothalamus reduces hypothalamic inflammation in patients with cancer-related anorexia, which leads to increased food intake to improve overall survival (35). Brain-derived neurotrophic factor (BDNF) reduces hypothalamic inflammation and increases sympathetic activation, thereby inhibiting cancer growth (36, 37). In addition, administration of an IL-1β receptor antagonist blocks the effects of a peripheral injection of cytokines (38, 39) and prevents anorexia in animal models of cancer (40).

Many studies have shown that intraventricular (ICV) IL-1β injections cause profound behavioral changes in rodents (19). The injection of central or peripheral cytokines indicates that proinflammatory mediators exert their effect primarily at the central level rather than the peripheral level (41, 42). Therefore, the brain is a more complex and advanced organ, and the action of cytokine signaling overrides the effect of peripheral signaling to maintain normal function. Some studies have shown that endogenous IL-1β expressed in the brain mediates lipopolysaccharide (LPS)-induced anorexia by modulating the expression of cytokines in the hypothalamus (43). Multiple circulating cytokines and inflammatory mediators trigger CNS immune signaling after LPS injection in a short period instead of sustained hypothalamic inflammation or sickness behavior. More importantly, these animals only experienced a very short period of mild anorexia after LPS administration, and food intake and body weight recovered to normal levels after 6 hours (44, 45). LPS-induced anorexia depends on its central inflammatory mechanisms, and the central role of peripheral LPS may be mediated by certain cytokines and/or receptors expressed in the brain (45, 46). Therefore, these studies support the hypothesis that the hypothalamus senses metabolic and inflammatory signals to fully regulate energy homeostasis and systemic inflammation.

### Inflammatory Signaling Affects Appetite-Related Peptides in the Homeostasis System

In LPS- or TNF-α-induced anorexia experimental models of acute inflammation, NPY expression in the hypothalamus is reduced, consistent with the observed decrease in food intake (47–49). Furthermore, the administration of NPY or blocking the induced inflammatory response prevents the development of anorexia (47, 50). Thus, NPY release is necessary to change food intake in a manner dependent on energy expenditure. However, NPY mRNA expression in the hypothalamus increases in animal models of chronic inflammatory diseases characterized by cachexia, such as cancer cachexia and arthritis (51–55). However, this increase in NPY mRNA levels did not correlate with a decrease in food intake (52, 54, 56, 57). It was associated with weight loss. In addition, NPY levels and NPY release are either unchanged (52) or decreased in these animal models (58–60). In these cases, NPY signaling is likely regulated at the posttranscriptional level. Therefore, we propose that NPY mRNA expression might represent a sensor for weight loss and the appropriate translation and release of NPY are subsequently required to determine changes in food intake and energy expenditure. The AgRP expression pattern is similar to NPY, as its mRNA expression increases (61) but secretion decreases in both acute and chronic inflammatory anorexia models. In addition, the changes in AgRP levels are associated with weight loss. In addition, genetic models of anorexia suggest that the disruption of NPY/AgRP signaling is partially due to axonal transport dysfunction (62). Altogether, these data indicate that inflammatory mediators affect NPY/AgRP mRNA expression in a posttranscriptional manner, including altered NPY translation, synthesis, packaging, or release, ultimately changing food intake.

Acute inflammation activates POMC neurons, which increase melanocortin-4 receptor (MC4R) expression (63) and POMC expression (64, 65), as shown in the LPS- and IL-1β-induced anorexia model. In addition, IL-1β binds to IL-1β receptor-expressing POMC neurons in the Arc of the hypothalamus to promote the effect of inflammation downstream (62). In fact, researchers have shown that the NF-κB pathway plays an important role in disease-induced anorexia and weight loss using different methods. Both the administration of AgRP and inhibition of the NF-κB pathway, especially in POMC neurons, significantly attenuates the effects of LPS on food intake and body weight. Therefore, POMC is a potential mediator of illness-induced anorexia and a downstream target of NF-κB. Interestingly, some researchers suggest that leptin-induced anorexia may also partially depend on the NF-κB pathway (64).

However, there are no obvious evidence that indicated the contribution of TLR4 in neurons or non-neuronal in the hypothalamus. On one hand, the activation of TLR4 signal in AgRP/NPY and POMC neurons decreased the expression of AgRP/NPY and increased the expression of POMC, which induced the reduction of appetite and suggested to be involved in obesity. On the other hand, the activation of TLR4 in non-neuronal cells, like microglia or astrocyte will increased the mRNA levels of specific inflammatory genes, which repression of TNF-α expression in the hypothalamic neurons (66). This evidence shown TLR4 in non-neuronal cells may affect the function of neurons in hypothalamus and potentially make a contribution of appetite and obesity.

## Appetite in a Non-Homeostatic State is Regulated by Inflammatory Signaling

### Increasing Inflammation Decreases Appetite

Consuming palatable foods is known to activate the reward system in the brain, which plays a critical role in appetite (67). The midbrain DA system has been suggested to play an important role in the regulation of reward-related behaviors (68, 69). DA neurons are located in the ventral tegmental area (VTA) and mainly project onto the nucleus accumbens (NAc) in the ventral striatum (70–73) and the prefrontal cortex (PFC) (73–76), which are associated with appetite. Although many factors influence appetite, inflammation is one of factors that should be considered (77, 78). Inflammation-induced sickness and behavioral changes are examples of inflammation altering motivational states (79). The host responds to the infection detected by innate immune cells and exhibits drastic behavioral changes, which facilitate the development of fever and fight against pathogens to maintain energy balance. Meanwhile, the results of many laboratory studies consistently show that innate immune system activation and release of inflammatory cytokines preferentially affect the reward circuit and basal ganglia DA level, leading to decreased appetite (80–82). Given the importance of DA in the reward system, a few studies have examined the relationship between inflammatory markers and symptoms/signs, which have shown an association between increased inflammation and decreased appetite (83–85).

These findings indicated that motivational symptoms like anergia and fatigue. Clinical data support the hypothesis that the effect of inflammation on appetite is driven by the action of cytokines on the DA system, as described below. In humans, much of the evidence is derived from the acute administration of inflammatory stimuli (such as endotoxin or typhoid vaccination) to healthy volunteers and the chronic administration of inflammatory cytokines [such as interferon alpha (IFN-α)] to patients as a treatment for certain cancers and infectious diseases (86, 87). These drugs induce the release of the inflammatory cytokines IL-6, IL-1β and TNF-α (88–90). IFN-α-treated patients have a reduced motivation to obtain food (86, 87). Neuroimaging studies have shown that the administration of inflammatory cytokines or cytokine attractants alters the activation of brain regions associated with appetite, including a decreased response to pleasurable food (82, 87, 91).

Initial neurochemical and behavioral studies reported that inflammation affects brain DA levels following acute or chronic injection of IFN-α in rodents. Based on these results, chronic administration of IFN-α decreases DA release in the striatum, which correlates with reduced appetite. In addition, long-term use of IFN-α reduces the reward associated with food but does not change sucrose consumption in monkeys. *In vivo* microdialysis was conducted to assess the effects of cytokines on the synaptic availability and release of striatal DA and to reveal the concrete effect of inflammation on appetite (92). Similar to the effect of IFN-α, peripheral administration of IL-1β to rodents also reduced appetite (85). Interestingly, endotoxin administration to rats reduces appetite but increases the motivation to obtain rest in a running wheel. Thus, the body will reduce energy consumption in order to survive in a pathological state.

A single intraperitoneal injection of LPS was administered to induce peripheral inflammation. Twenty-four hours after LPS administration, mice showed reduced appetite. Meanwhile, acute low-dose LPS (100 g/kg) systemic administration reduced the DA content in the NAc and increased extracellular DA metabolite levels (93, 94). As a result, LPS reduces incentive motivation for food rewards (95). The acute and chronic effects of LPS on brain DA levels are blocked by inhibiting or deleting genes encoding inflammatory cytokines such as TNF-α (96, 97). Inflammation-related medical disease models, such as experimental tumors, are associated with reduced brain DA levels (98, 99). Together, these results from animal studies suggest that various inflammatory stimuli consistently affect DA levels in the brain, leading to related changes in appetite. The DA precursor levodopa (L-DOPA) completely reverses IFN-α-induced reductions in striatal DA release observed using *in vivo* microanalysis, potentially indicating that cytokines may reduce DA synthesis (100). The effects of inflammation on DA and neural activation and metabolism in the reward circuit were examined, and IFN-α-treated subjects were administered [18F] fluorodopa (FDO-PA) (87). L-DOPA is absorbed by DA neurons and converted to DA by DA decarboxylase, which is then stored in vesicles and released. Interestingly, patients exhibited increased absorption and reduced turnover of L-DOPA in the ventral striatum after IFN-α treatment.

Our previous study has reported that TLR4 in dopamine neurons affect the food reward (food motivation) and food preference (HFD consumption) at the same time. In this process, deletion of TLR4 in dopamine neurons decreased the dopamine level in the brain, which contributes to the reduction of HFD preference. However, global TLR4 KO mice decreased expression of the key taste molecules cluster of differentiation 36 (CD36), phospholipase Cβ2 (PLCβ2) and transient receptor potential cation channel, superfamily M, member 5 (TRPM5) in tongue epithelium, which induced the change of food preferences. Therefore, TLR4 signaling in epithelium still can affect the food preference by decreasing the nutrient sensing (101).

### The Mechanism by Which Inflammation Regulates Appetite

**Figure 2:**
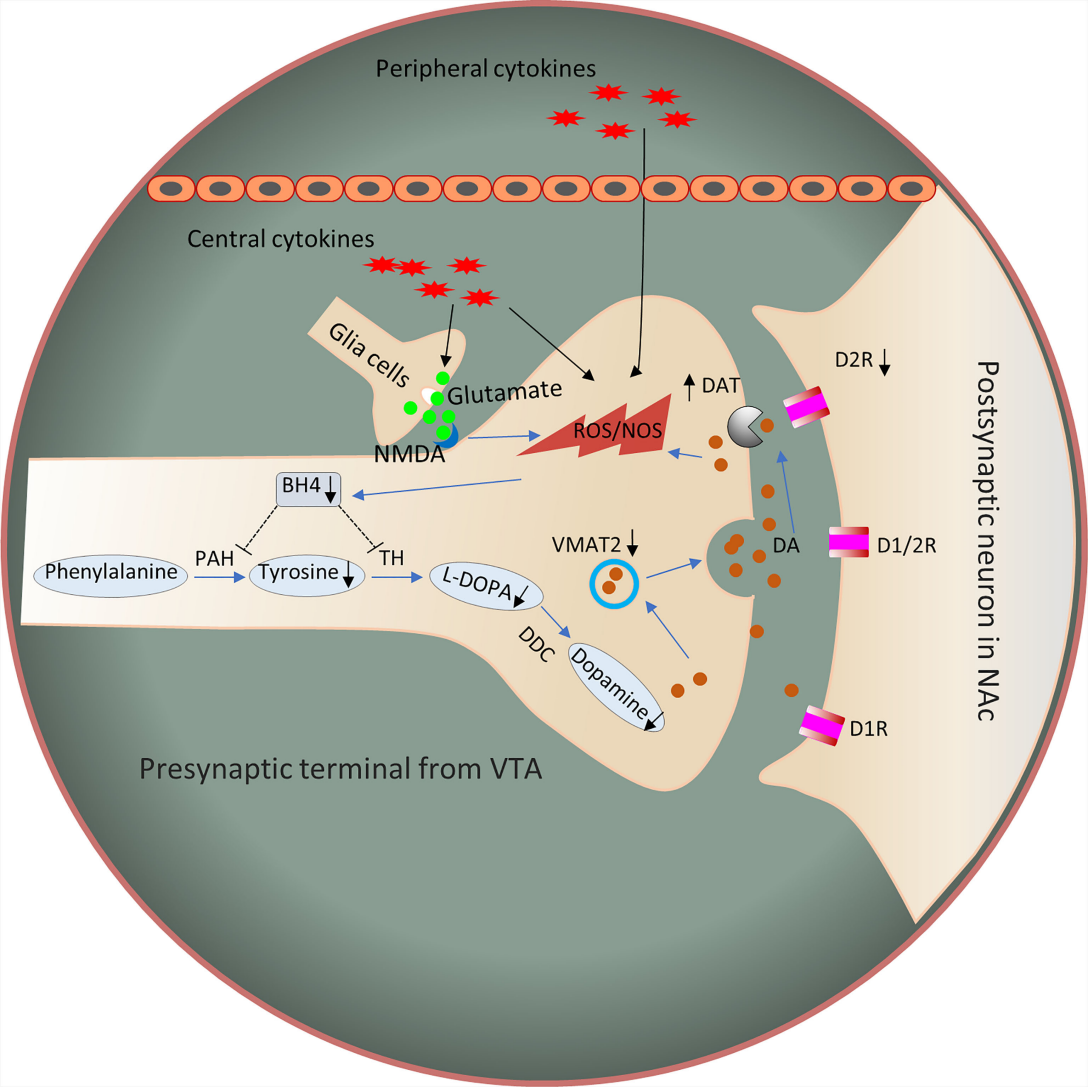
Potential mechanisms by which inflammation affects DA signaling through synthesis, release, and receptor function. Inflammation and cytokines released from the periphery or produced locally by activated microglia or infiltrating macrophages contribute to oxidative stress and the production of reactive oxygen species (ROS). Increased ROS and inflammation-induced nitric oxide levels contribute to the oxidation of BH4, an essential cofactor required for the conversion of phenylalanine to tyrosine and tyrosine to L-PODA, which are necessary for the synthesis of DA. In addition, the increased glutamate release and reduced reuptake by inflammation-induced glial cells, coupled with the activation of NMDARs, may cause excitotoxicity of glutamate. In turn, these changes lead to oxidative stress and decreased DA availability. Furthermore, some evidence has shown that inflammatory cytokines reduce the expression or function of VMAT2 and/or increase the expression or function of DAT and reduce DA signaling by reducing the levels of DA D2 receptors. Dysregulation of DAT and VMAT2 increases cytosolic DA levels, leading to auto-oxidation and ROS generation. D1R, dopamine 1 receptor; D2R, dopamine 2 receptor; DDC, dopamine decarboxylase; NMDAR, N-methyl-D-aspartic acid receptor; NOS, nitric oxide synthase; ROS, reactive oxygen species; PAH, phenylalanine hydroxylase; TH, tyrosine hydroxylase; BH4, 5,6,7,8-tetrahydrobiopterin; VMAT2, vesicular monoamine transporter 2; LPODC, L-3,4-dihydroxyphenylalanine.

In summary, evidence from humans and animals suggests that inflammation is associated with the reduced DA availability and release, which exert functional effects on reward circuits. These changes are associated with fundamental changes in motivation and motor function. The mechanisms underlying the effects of cytokines on DA synthesis, release, reuptake, or receptor signaling should be considered to determine the changes in the DA system in the inflammatory process that correlate with reward and motivation. Inflammation and cytokines affect DA-related functions through multiple pathways. DA synthesis, packaging and release, reuptake and DA receptors may interact to reduce the DA signal in the basal ganglia. Therefore, the next sections mainly discuss the potential mechanisms by which inflammation affects DA neurotransmission (**Figure 2**).

#### DA Synthesis and Availability

DA synthesis relies on tyrosine hydroxylase (TH), which is the rate-limiting enzyme required for DA synthesis to convert tyrosine to L-DOPA. Phenylalanine hydroxylase (PAH) is an enzyme that converts phenylalanine to tyrosine. 5,6,7,8-Tetrahydrobiopterin (BH4) is a cofactor of aromatic amino acid monooxygenases. Both TH and PAH require the enzyme cofactor BH4 (102). Inflammation may decrease BH4 availability (103). Indeed, intramuscular administration of IFN-α in rats has been shown to reduce the concentration of BH4 in the CNS by stimulating nitric oxide (NO), while inhibition of NO reverses the inhibitory effect of IFN-α on brain concentrations of BH4 and DA (84). In addition, the administration of IFN-α also increases cerebrospinal fluid (CSF) IL-6 levels, which were also correlated with decreased BH4 levels in CSF (92). Evidence from IFN-α-treated patients has also revealed a reduction in BH4 levels (104). In addition, the peripheral blood phenylalanine/tyrosine ratio increases after IFN-α administration, which correlates with decreased DA synthesis and CSF levels of DA and its major metabolite HVA (92). However, no change was observed in the DOPAC/DA ratio after L-DOPA administration, which increases when DA is not packaged and secreted in synaptic vesicles and subsequently metabolized by monoamine oxidase (105). These findings are consistent with decreased levels of DA metabolites in the CSF of IFN-α-treated patients and monkeys (88, 106). Meanwhile, L-DOPA reverses IFN-α-induced reduction in DA release (100). Notably, IL-6 treatment has also been shown to reduce the BH4 content in sympathetic neurons (107).

Another mechanism may be that cytokines affect glutamate neurotransmission to change the function of the basal ganglia and DA. In addition, inflammatory cytokines increase extracellular glutamate levels by reducing the level of the excitatory amino acid transporter GLT-1 (increasing glutamate reuptake) and increasing glutamate release from astrocytes and activated microglia (108, 109). Then, glutamate binds to the N-methyl-D-aspartic acid receptor (NMDA) and potentially leads to excitotoxicity in the brain (110). As a result, oxidative stress increases and potentially contributes to the effects on BH4 and DA synthesis (106, 111).

#### DA Packaging, Release and Reuptake

In synapses, vesicular monoamine transporter 2 (VMAT2) packages cytoplasmic DA into vesicles for further release. The inflammatory cytokines IL-1β and TNF-α have been shown to decrease the expression of VMAT2 in rat enterochromaffin-like cells, whereas the administration of transforming growth factor-β (TGF-β), which is an immunomodulatory and anti-inflammatory growth factor, rescues VMAT2 expression (112). Therefore, inflammatory cytokines and inflammation may negatively affect VMAT2 expression and function. In addition, the administration of pituitary adenylate cyclase-activating polypeptide 38 (PACAP-38), an anti-inflammatory compound, *in vivo* reduces neuroinflammation and increases VMAT2 expression, which protects against DA neurotoxicity following chronic methamphetamine exposure (113). IFN-α and other cytokines activate p38 mitogen-activated protein kinase (MAPK) signaling, which plays an important role in the expression and function of the serotonin transporter in serotonin reuptake (114, 115). Recently, researchers have found that MAPK pathways also influence DAT (DA transporter). DAT is expressed in DA neurons, and the role of DAT is to induce DA reuptake by DA neurons after its release into synapses. DAT-expressing neurons transfected with activated MAPK kinase (MEK) exhibit increased DA reuptake. Administration of MEK inhibitors to rat striatal synaptosomes decreases DA reuptake in a dose- and time-dependent manner (116). Therefore, exposure to inflammatory cytokines reduces synaptic DA levels through a mechanism that may be associated with increasing DAT expression or function.

#### DA Receptor Expression and Function

Type D2 dopamine receptor (D2R) is a G-protein-coupled receptor located in postsynaptic dopaminergic neurons that is mainly involved in reward mediation and reward deficiency pathways (117). A recent study elegantly showed that D2R within the ventrolateral striatum plays an important role in motivated behavior (118). Conditional knockout of D2R reduces the progressive rate task breakpoint, while optogenetic inhibition of these neurons that express D2R causes a transient reduction in the breakpoint. Inflammation and cytokines may affect DA signaling by reducing the expression or function of DA receptors. Chronic administration of IFN-α in the striatum of monkeys decreases the binding of DA to D2 receptors (83).

## Indirect Mechanism of TLR4 Expression in the Perphery

Although TLR4 expression in CNS plays an important role in eating disorder, the TLR4 in periphery still affects the process of appetite. In activity-based anorexia (ABA) model mice, the expression of TLR4 in colonic mucosa is higher. Meanwhile, the mucosal cytokines expression also increased during ABA mice. Interestingly, TLR4, MyD88, TLR adaptor molecule 1 (TRIF) and TRIF-related adaptor molecule (TRAM) remained unchanged in the hypothalamus, but increased the expression of IL-1β, IL-1β receptor 1 (IL-1R1) and Interleukin-1 receptor-associated kinase (IRAK-4) in hypothalamic (119). The indirect mechanisms may be the peripheral cytokines cross the blood-brain barrier and activate neuro-inflammation in the brain. In addition, another paper reported that global TLR4 KO mice decreased the expression of TLR4 in tongue gustatory epithelium, which may affect expression of taste molecules CD36, PLC2β and TRPM5 and change food intake (101).

## The Role of TLR4 Activation in the Regulation of Appetite

As an innate immune receptor, TLR4 is well known for its response to LPS. However, it is also activated by nutritional signals, such as saturated fatty acids (SFAs), particularly lauric acid, palmitic acid, and stearic acid (120). TLR4-dependent priming senses the lcSFA and regulates gene expression and cellular metabolism (121). Chronic overconsumption of a HFD can increase the plasma levels of free fatty acids (122). Increased fatty acid concentrations in plasma are closely associated with metabolic syndrome, and SFAs activate innate immune responses and result in inflammation (123–125).

SFAs have been shown to cause hypothalamic inflammation (126), and chronic hypothalamic inflammation disrupts the function of brain circuits that control appetite, leading to an increase in food intake and weight gain (127). The dietary lipid composition will affect the degree of inflammation in the hypothalamus. For example, a diet high in saturated fat is associated with a higher risk of hypothalamic inflammation after 8 weeks than a diet high in unsaturated fat (128). In addition, fat from butter produces greater neuroinflammation than saturated fat from coconut oil, indicating that fat from different sources produces different hypothalamic inflammatory responses. SFAs exert major effects on neuro-immunity, enteroendocrine signaling, feeding homeostasis and appetite regulation. In addition, some studies attribute hypothalamic inflammation to SFAs. In this process, inflammatory signaling is activated by the SFA-TLR4 pathway. These free fatty acids bind to receptors on immune cells, activate inflammatory signaling pathways, and impair normal cellular signaling in the liver, pancreas, skeletal muscle, and white adipose tissue (129), resulting in the release of proinflammatory cytokines and chemokines.

Based on these findings, SFAs can activate TLR4 signal pathway, which plays an important role in physiological regulation of brain function (130, 131). SFA activates immune cells, including microglia, through TLR4 signal pathway (11). In the brain, palmitic acid activates microglia to release cytokines *via* the TLR4-activated signal transduction pathway, which leads to a decrease in hypothalamic cell activity, thus leading to the interruption of the neural circuit controlling appetite (132). Lauric acid, palmitate and stearic acid increase the release of TNF-α and IL-6 from astrocytes. Apparently, the increase in TNF-α and IL-6 levels in the hypothalamus mediates insulin and leptin resistance by increasing the level of the suppressor of cytokine signaling 3 (SOCS3) protein and phosphorylation of insulin resistance substrate and suppressing the Janus kinase/signal transducer and activator of transcription (JAK-STAT) pathway, which induces leptin receptor activation. In addition, the effect of palmitic acid on cytokine release requires TLR4 rather than CD36 or toll like receptor 2 (TLR2) (which are also palmitic acid receptors) and is independent of palmitate metabolism to palmitoyl-CoA (133). Accordingly, short-term ICV administration of stearic acid promotes TLR4 activation and the expression of endoplasmic reticulum (ER) stress-related proteins, while unsaturated fatty acids attenuate the inflammatory response. Stearic acid elicits AgRP expression and secretion *via* TLR4-dependent signaling pathways in hypothalamic N38 cells (134). However, the result has not been verified in animals. Thus, LCSFAs play a main role in hypothalamic inflammation and appetite (135). Additionally, chronic hypothalamic inflammation can also lead to leptin and insulin resistance, which will further weaken the homeostatic signaling of the hypothalamic circuit. However, evidence for the mechanism by which SFAs affect motivation in humans and animals is lacking. Previous studies reported that a single intra-VTA injection of palmitic acid does not affect food intake (136), but lauric acid increases appetite in mice (137). The proinflammatory state of glial cells is activated by SFAs *via* TLR4/NF-kB signaling in a microglial cell line. Therefore, SFAs activate TLR4 signaling, which is an important process for understanding how SFA-induced inflammation regulates appetite.

## Conclusion

**Figure 3:**
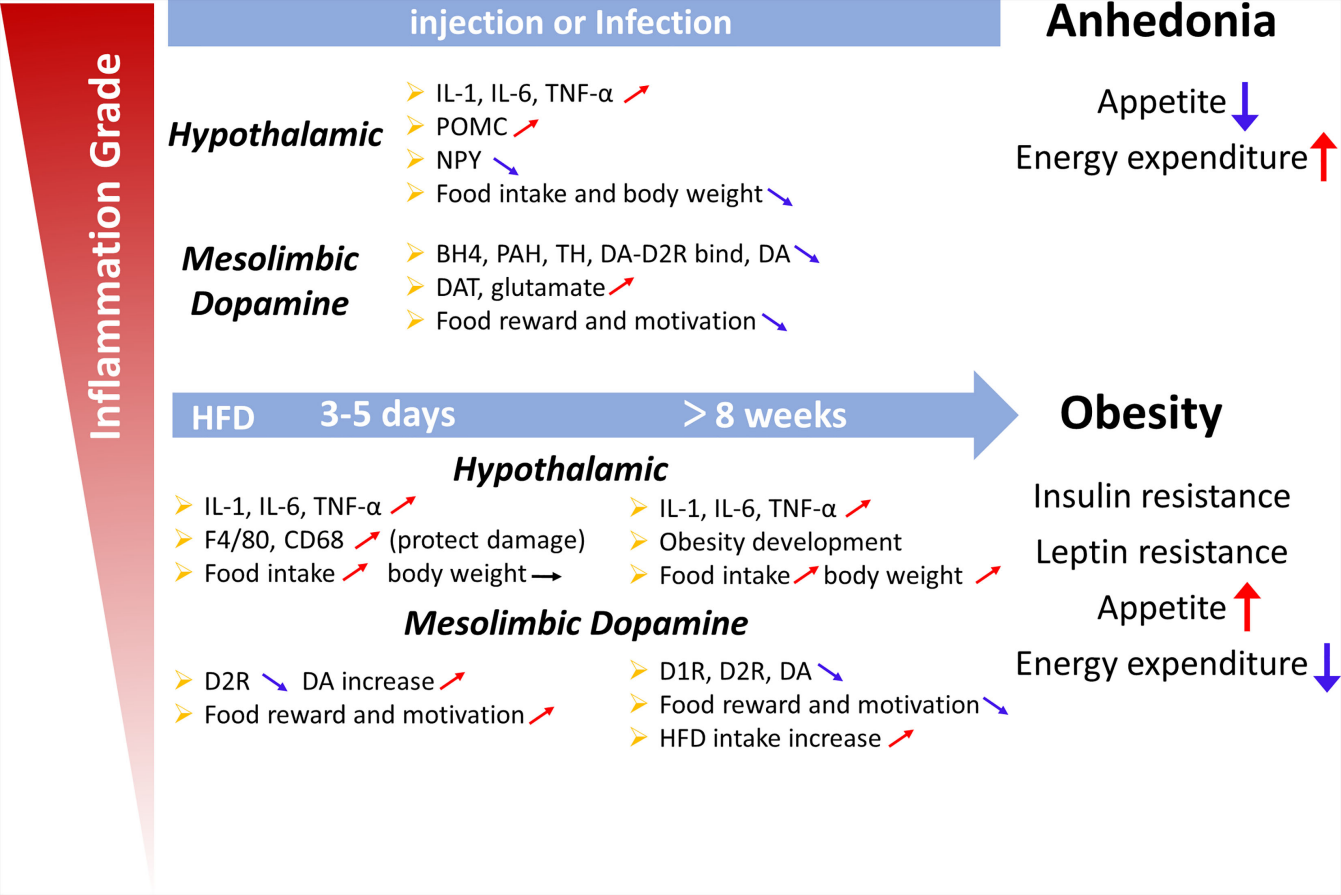
Summary diagram illustrating the links between inflammation in the hypothalamus and midbrain dopamine system with the deregulation of appetite. Upper panel: Lipopolysaccharide or cytokines injections, illnesses such as cancer, or infections induce high-grade inflammation. An acute increase in the local production of cytokines and chemokines is observed in the hypothalamic and midbrain dopamine systems. Cytokines, including IL-1, IL-6 and TNF-α, change the levels of neuropeptides (POMC, NPY and dopamine) involved in the hypothalamic and dopamine systems, which are associated with appetite and weight loss (the sickness behavior associated with a high-intensity infection). Lower panel: Short (3 days) or chronic (>8 weeks) HFD consumption is associated with low-grade inflammation. The increase in cytokine levels induced by a HFD also changes the levels of neuropeptides involved in the hypothalamic and dopamine systems, which appears to be associated with overeating and body weight gain and the development of obesity, leptin resistance and insulin resistance, potentially increasing appetite, and weight. However, in the first 3 days, the weight was not changed, which may protect organs from HFD-induced damage.

In this review, we showed that inflammatory signals in the hypothalamus and mesolimbic DA system play different roles in regulating appetite. In summary, cytokine release induced by inflammatory signals decreases NPY and AgRP expression and increases POMC expression to decrease food intake. In addition, inflammatory cytokines may affect multiple aspects of DA neurotransmission, resulting in reduced synthesis and impaired DA receptor signaling and/or packaging or release, all of which interact to reduce DA function, which contributes to appetite (**Figure 3**). As ligands, SFAs also trigger inflammation in the brain though TLR4 to affect appetite.

## Perspectives and Future Directions

Appetite is controlled by several complex regulatory mechanisms involving both homeostatic and non-homeostatic processes. Additionally, inflammation is seen as a way for tissues to try to return to normal in response to infection or disruption. Such studies have provided evidence in understanding the full impact of TLR4-induced inflammatory signaling on appetite and how it may increase the risk of developing obesity and related health problems. Importantly, the interfering of appetite can be achieved by regulating inflammatory signaling in hypothalamus and dopamine system, which could guide development of novel therapies to treat diseases about appetite. More importantly, the link between SFAs and CNS immune system also provides an exciting new direction for the study of eating behaviors and the pathophysiology of obesity.

## Author Contributions

YL wrote the manuscript. QJ provided constructive comments for the figure drawing. LW revised the manuscript. All authors contributed to the article and approved the submitted version.

## Funding

This work was supported by the National Natural Science Foundation of China (32072779).

## Conflict of Interest

The authors declare that the research was conducted in the absence of any commercial or financial relationships that could be construed as a potential conflict of interest.

## Publisher’s Note

All claims expressed in this article are solely those of the authors and do not necessarily represent those of their affiliated organizations, or those of the publisher, the editors and the reviewers. Any product that may be evaluated in this article, or claim that may be made by its manufacturer, is not guaranteed or endorsed by the publisher.
